# Concurrent chronic lymphocytic leukemia and primary hyperparathyroidism in a mule

**DOI:** 10.1111/jvim.16714

**Published:** 2023-04-28

**Authors:** Kile S. Townsend, Philip J. Johnson, Lindsay L. Donnelly, Alison M. LaCarrubba, James C. Lattimer, Brett Havis, Nora L. Springer, Dae Y. Kim

**Affiliations:** ^1^ Department of Veterinary Medicine and Veterinary Medical Diagnostic Laboratory College of Veterinary Medicine, University of Missouri Columbia Missouri USA; ^2^ Department of Biomedical and Diagnostic Sciences University of Tennessee Knoxville Tennessee USA

**Keywords:** endocrinopathy, equine, hypercalcemia, immunohistochemistry, neoplasia

## Abstract

A 26‐year‐old mule gelding was evaluated for chronic weight loss and decreased appetite. The mule had been losing weight and intermittently hypophagic for approximately 7 months. Laboratory analysis of whole blood and plasma identified severe total hypercalcemia, marked hypophosphatemia, markedly increased parathyroid hormone concentration, and marked lymphocytosis. A sestimibi scan intended to identify parathyroid gland tissue was nondiagnostic. Results of flow cytometry and PCR for antigen receptor rearrangement (PARR) were consistent with a B cell lymphoproliferative disorder, likely chronic lymphocytic leukemia (CLL). Although not previously described concurrently, these conditions may sometimes arise together, complicating definition of the underlying mechanism for weight loss and hypercalcemia in aged equids.

**Abbreviations**
ACTH: adrenocorticotropic hormone
AST: aspartate aminotransferase
BCS: body condition score
CD: cluster designation
CLL: chronic lymphocytic leukemia
GGT: γ‐glutamyl transferase
EOTRH: equine odontoclastic tooth reabsorption and hypercementosis
HE: hematoxylin and eosin
MHC: major histocompatibility complex
PTH: parathyroid hormone
PTHrP: parathormone related protein
PARR: PCR for antigen receptor rearrangement
PCR: polymerase chain reaction
PBP: plasma biochemistry profile
PHPT: primary hyperparathyroidism
PPID: pituitary pars intermedia dysfunction
RBC: red blood cell
RDVM: referring veterinarian
RI: reference interval
SLL: small lymphocytic lymphoma
^99m^Tc‐sestamibi: Technetium99m (^99m^Tc)‐sestamibi
VHC: Veterinary Health Center

## 1 CASE DESCRIPTION

### 1.1 Initial presentation

A 25‐year‐old mule gelding was referred to the University of Missouri Veterinary Health Center for weight loss and inappetence of 7 months' duration. Hypercalcemia had been identified 3 months earlier. Plasma adrenocorticotropic hormone (ACTH) concentration was not increased, and fecal parasitic ova were not evident at that time. An agar gel immunodiffusion assay (Coggins test) for equine infectious anemia was negative. Weight loss progressed despite dental rasping, deworming, and dietary enhancements. Appetite continued to worsen throughout this time, but no other clinical signs were reported.

At admission, the gelding was alert and responsive. Rectal temperature (36.6°C), heart rate (48 beats per minute [bpm]), and respiratory rate (28 breaths per minute) were normal. The gelding was thin (body condition score [BCS], 3/9; weight, 422 kg).^1^ Moderate halitosis was attributed to equine odontoclastic tooth reabsorption and hypercementosis (EOTRH). Cardiopulmonary auscultation was normal.

Hematological abnormalities included mild leukocytosis (14.60 × 10^3^ cells/μL; reference interval [RI], 4.79‐10.88 × 10^3^ cells/μL), mild mature neutrophilia (7.15 × 10^3^ cells/μL; RI, 2.4‐6.27 × 10^3^ cells/μL), and mild lymphocytosis (7.15 × 10^3^ cells/μL; RI, 1.15‐4.58 × 10^3^ cells/μL) with a few reactive lymphocytes. Plasma biochemistry profile (PBP) abnormalities included marked total hypercalcemia (22.2 mg/dL; RI, 11.2‐12.8 mg/dL), marked hypophosphatemia (<1.0 mg/dL; RI, 1.8‐4.0 mg/dL), mild hypermagnesemia (2.5 mg/dL; RI, 1.5‐2.3 mg/dL), and mildly increased aspartate aminotransferase (AST; 549 U/L; RI, 203‐415 U/L) and γ‐glutamyl transferase (GGT; 40 U/L; RI, 10‐30 U/L) activities. Results of urinalysis were normal.

Endocrine testing included assessment of plasma parathyroid hormone (PTH), ionized calcium (iCa), parathormone‐related protein (PTHrP), and 25‐hydroxyvitamin D concentrations. Diagnosis of primary hyperparathyroidism (PHPT) was based on increased plasma PTH (15.5 pmol/L; RI, 0.6‐11.0 pmol/L) and markedly increased ionized calcium (3.41 mmol/L; RI, 1.58‐1.90 mmol/L) concentrations (measured at the Michigan State University Veterinary Diagnostic Laboratory, employing a validated whole‐molecule immunoradiometric assay).^2^ Concentrations of PTHrP and 25‐hydroxyvitamin D were normal.

Results of thoracic and abdominal ultrasonography, gastroesophageal endoscopy, and abdominocentesis were normal. Radiography of the incisors confirmed EOTRH.

Further diagnostic tests were declined and the gelding was pasture accommodated. Thirteen months later, ongoing loss of condition led to donation of the mule.

### 1.2 Second presentation

The gelding was bright and alert. Rectal temperature (36.7°C) was normal but heart (56 bpm) and respiratory (40 breaths per minute) rates were increased. The mule was still thin (BCS, 3/9) and weight had slightly decreased (408 kg). The EOTRH was unchanged. Symmetrical lymph node enlargements were evident, especially in the mandibular and inguinal regions. Bilaterally symmetrical, firm, ~10 cm spherical masses were palpated in the pre‐scapular region.

Laboratory abnormalities included hyperproteinemia (8.2 g/dL; RI, 6.1‐7.5 g/dL), mild anemia (RBC, 4.92 × 10^6^/μL; RI, 6.41‐10.12 × 10^6^/μL; hemoglobin concentration, 10.3 g/dL; RI, 11.4‐16.9 g/dL), and marked leukocytosis (65.73 × 10^3^/μL) because of mild neutrophilia (6.57 × 10^3^/μL) and marked lymphocytosis (58.17 × 10^3^/μL). Lymphocytes were characterized as small to intermediate‐sized with slightly round to ovoid and sometimes focally indented eccentric nuclei of ~1.25 to 2 RBC diameters in size, with coarsely stippled to clumped chromatin, indistinct nucleoli, and small amounts of lightly basophilic cytoplasm, sometimes containing fine magenta‐colored granules. Lymphocyte appearance suggested either chronic lymphocytic leukemia (CLL) or a leukemic phase of lymphoma.

Clinically relevant PBP abnormalities included hyperproteinemia (8.0 g/dL; RI, 5.7‐7.5 g/dL), hyperglobulinemia (5.5 g/dL; RI, 2.4‐4.1 g/dL), marked hypercalcemia (25.4 mg/dL), marked hypophosphatemia (<1.0 mg/dL), hypermagnesemia (2.6 mg/dL), and mildly increased AST (667 U/L) and GGT (57 U/L) activities. Urinalysis indicated hyposthenuria. Fractional urinary excretion of calcium was low (2.07%; RI, 5.3%‐14.5%). Serum protein electrophoresis disclosed mildly increased alpha‐1 and gamma fractions, with a broad‐based, polyclonal increase (suggestive of mild acute‐on‐chronic inflammation).

Plasma ACTH concentration (26.5 pg/mL) did not indicate pituitary pars intermedia dysfunction (PPID) (seasonal cut‐off, <35 pg/mL). Plasma PTH concentration was still markedly increased (25.0 pmol/L) with ionized hypercalcemia (4.19 mmol/L), but PTHrP, 25‐hydroxyvitamin D, and calcitriol concentrations were normal, consistent with the previous diagnosis of PHPT.

Further testing for lymphoproliferative neoplasia included abdominothoracic and cervical ultrasonography, peritoneal fluid analysis, and fine needle aspiration of cervical lymph nodes. Ultrasonography disclosed increased peritoneal fluid and mesenteric lymphadenomegaly. Peritoneal fluid nucleated cell count and protein concentration were normal; presence of lymphocytes similar to those seen in blood suggested that they likely resulted from lymphoproliferative neoplasia.

Enlarged cervical lymph nodes were ultrasonographically characterized by a mixed echogenic pattern. Aspirates of the right mandibular node and the lymph node at the right thoracic inlet were interpreted as reactive lymphoid hyperplasia. However, small cell lymphoma with leukemia was not entirely discounted.

wA flow cytometric immunophenotyping panel (Table 1) was performed at the Kansas State Veterinary Diagnostic Laboratory. The gated lymphocyte population had positive labeling for CD44 (91%) and CD21 (66%). A small proportion of lymphocytes labeled positively for CD5+ (8%), CD4+ (6%), and CD8+ (<1%). Approximately 28% uncharacterized cells were CD4 negative, CD8 negative and CD21 negative. The laboratory‐specific reference ranges for healthy adult horses are CD4+ (53%‐79%), CD8+ (13%‐20%), and B‐cell (5%‐16%). Cluster of differentiation antigen‐21 is a B cell marker whereas CD5, CD4, and CD8 are T cell markers. When correlated to the blood smear findings, the immunophenotyping interpretation was a B cell lymphoproliferative disorder, small lymphocyte lymphoma (SLL) or CLL. Based on prolonged lymphocytosis and cell morphology, this presentation is most consistent with CLL. Results of PCR for antigen receptor rearrangement (PARR) of an enlarged lymph node identified clonal rearrangements of B cell genes and polyclonal rearrangements of T cell genes, which also was consistent with B cell lymphoproliferative disease.^3^

**TABLE 1:** Table depicting antibody clone, manufacturer, and target for the flow cytometric immunophenotyping panel and immunohistochemistry (IHC).

Marker	Clone	Manufacturer	Primary target
CD44	IM7	BioLegend	Pan‐leukocyte
CD5	CD5/54/F6	Novus Biologicals	T lymphocytes
CD4	CVS4	Bio‐Rad	T helper cells
CD8	CVS21	Bio‐Rad	Cytotoxic T cells
CD21	CA2.1D6	Bio‐Rad	B lymphocytes
CD3 (IHC)	A0452	DAKO	T lymphocytes
CD20 (IHC)	ab64088	Abcam	B lymphocytes

A sestamibi parathyroid scan was performed to locate 1 or more parathyroid gland tumors. Technetium99m (^99m^Tc)‐sestamibi (925 MBq [25 mCi]) was injected IV and ventral imaging of the neck and thoracic inlet region was performed immediately with acquisition times of 4 to 7 minutes. Additional images were acquired at 2 and 4.5 hours post‐injection. Normal uptake of the radioisotope preparation in the salivary glands was observed indicating that preparation of the isotope was appropriate. No uptake was observed around the thyroid or parathyroid glands. Thoracic inlet scanning did not indicate any uptake. The gelding was euthanized and necropsy performed.

## 2 NECROPSY FINDINGS

Enlargement of multiple lymph nodes throughout the body was the most prominent gross pathological finding. Affected lymph nodes included the retropharyngeal, mandibular, prescapular, cranial mediastinal, tracheobronchial, axial, mesenteric, iliac, and inguinal lymph nodes; the largest lymph node (mesenteric) measured 7 × 5.5 × 3 cm. Affected lymph nodes varied in color with some being diffusely tan to white and others having a mottled red and tan appearance. Many enlarged lymph nodes were present in proximity to the thyroid gland and identification of the parathyroid glands was not possible. Enlargement of the pituitary gland as well as EOTRH were also evident.

**FIGURE 1:**
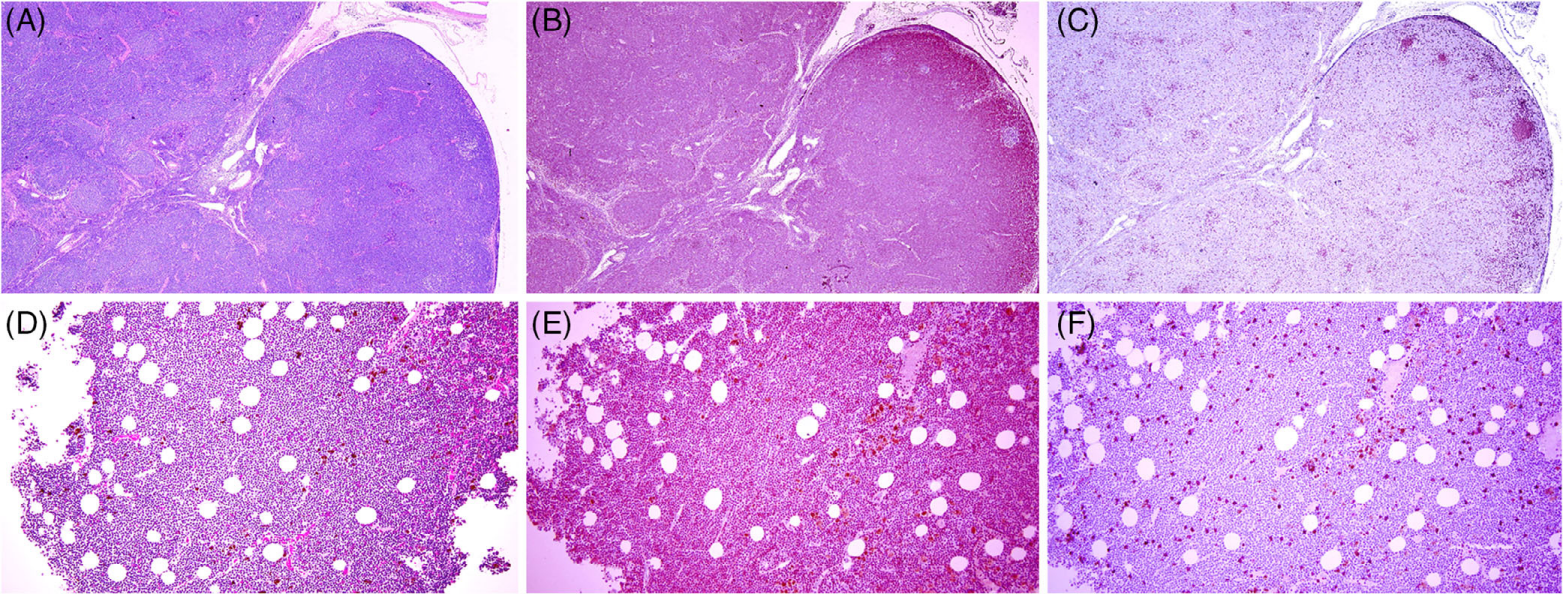
Chronic lymphocytic leukemia of a mule. (A) Histopathological appearance of a representative mandibular lymph node depicting diffuse infiltration by small round cells in coalescing nodules in the cortex and medulla, HE (10× magnification). (B) Immunohistochemical appearance of a representative mandibular lymph node depicting that the majority of the round (neoplastic) cells were red‐stained CD20‐positive B cells (10× magnification). (C) Immunohistochemical appearance of a representative mandibular lymph node showing that round (neoplastic) cells were negative for CD3 but that there were a few randomly scattered small aggregates of red‐stained CD3‐positive (inflammatory) T cells (10× magnification). (D) Histopathological appearance of bone marrow depicting a multifocally hypercellular pattern, HE (20× magnification). (E) Immunohistochemical appearance of bone marrow depicting that the majority of cells were red‐stained CD20‐positive B cells (20× magnification). (F) Immunohistochemical appearance of bone marrow depicting negative CD3 staining of neoplastic cells but that there were a few scattered red‐stained CD3‐positive (inflammatory) T cells (20× magnification). Romulin Red chromogen (Biocare) was used for the positive immunolabel and hematoxylin was the counterstain for immunohistochemical staining.

Microscopically, affected lymph nodes were infiltrated by a highly cellular, unencapsulated, poorly demarcated population of small round cells (1‐1.5 × the diameter of an erythrocyte) arranged in variably sized, dense, and loose aggregates. Neoplastic cells were characterized by distinct cellular margins with scant to small amounts of eosinophilic cytoplasm. Nuclei were round to oval with dense to coarsely stippled chromatin. Anisocytosis and anisokaryosis were mild and 13 mitotic figures per 2.37 mm^2^ were observed. Infiltrating neoplastic cells effaced the cortex, paracortex, medulla, and sinuses of affected nodes and infiltrated the fibrous capsule and surrounding adipose tissue (Figure 1). Bone marrow spaces were effaced by the same neoplastic cells, similarly arranged in variably sized, dense, and loose aggregates (Figure 1). Bone trabeculae were unremarkable. Infiltrating neoplastic cells also were observed in the portal veins and periportal sinusoids of the liver, pulmonary arteries, and in the lamina propria and submucosa throughout the gastrointestinal tract. Neoplastic cells were positive for CD20 but were negative for CD3 by immunohistochemistry (Table 1; Figure 1). Neoplastic cells in multiple lymph nodes were immunonegative on PTH immunohistochemistry. Results of immunohistochemistry performed on tissues at necropsy agreed with flow cytometry results (circulating lymphocytes). Histopathological diagnosis was B cell small cell lymphoma or CLL. Affirmative identification of parathyroid gland tissue was not achieved.

## 3 DISCUSSION

Development of both PHPT and CLL in the same equid has not been reported previously. As initially encountered in this mule, hypercalcemia is an uncommon clinical finding in equine clinical practice. Previously reported explanations for hypercalcemia in this species include PHPT, renal failure, vitamin D toxicosis, and cancer (paraneoplastic phenomenon).^4^ The initial diagnosis of PHPT was based on increased serum PTH concentrations and normal results for plasma PTHrP, vitamin D, creatinine, and urea nitrogen concentrations.

Although subtle lymphocytosis was noted initially, additional diagnostic tests were not performed. Thirteen months later, PHPT was still evident but worsening lymphocytosis, generalized lymphadenopathy, and hyperglobulinemia suggested concurrent lymphoproliferative neoplasia. Although hypercalcemia of malignancy has been associated with lymphoma, multiple myeloma, ameloblastoma, mesenchymal ovarian cancer, squamous cell carcinoma, gastric carcinoma, and adrenocortical carcinoma in horses,^5^, ^6^ failure to identify increased PTHrP concentrations or areas of malignancy‐associated osteolysis suggest that hypercalcemia resulted from PHPT. Moreover, PTH expression by neoplastic lymphocytes was considered but PTH immunohistochemistry on neoplastic cells was negative. Results of protein electrophoresis suggested chronic inflammation rather than excessive paraprotein production as described in horses with multiple myeloma or lymphoma.^7^

Hyperparathyroidism is uncommon in equids and can be primary or secondary.^8^, ^9^, ^10^, ^11^, ^12^ Whereas secondary hyperparathyroidism results from dietary factors that interfere with the oral bioavailability of calcium, renal secondary hyperparathyroidism have not been reported in this species.^13^, ^14^ Primary hyperparathyroidism results from parathyroid gland hyperplasia or adenoma, with the chief cells of ≥1 affected parathyroid glands secreting excessive PTH and dysregulating calcium and phosphorus homeostasis.^8^, ^9^, ^11^ Increased PTH concentration results in increased osteoclast activity, osteoid resorption (osteopenia), increased renal calcium resorption, and urinary phosphorus wasting.

Diagnosis of PHPT in horses should be suspected if hypercalcemia and hypophosphatemia are identified in the absence of renal disease and cancer. As with this mule, affected equids commonly develop weight loss and inappetence. In other cases, lameness and headshaking may arise because of stimulated bone resorption. Diagnostic affirmation is obtained using endocrine testing (increased PTH concentration in the absence of increases in PTHrP, vitamin D, and creatinine concentrations). Hyperparathyroidism may be discovered incidentally because some affected equids do not exhibit clinical signs over many years.^15^ This mule likely was affected for >2 years with only mild weight loss as the principal concern. Moreover, CLL, EOTRH, advancing age, and PPID probably contributed to the weight loss.

If a solitary adenomatous parathyroid gland is identified, surgical excision (parathyroidectomy) can be curative.^9^, ^11^ Recently, percutaneous ultrasound‐guided ethanol ablation was described as an alternative treatment.^16^ However, treatment success is limited by the fact that positive identification of an affected parathyroid gland is challenging in horses. As with humans and dogs, horses have 2 pairs of parathyroid glands. Being located anywhere along the length of the carotid arteries from the cranial cervical region to the thymus makes detection of a parathyroid adenoma affecting caudal parathyroid glands challenging using ultrasonography alone.^8^, ^9^

The sensitivity of ^99m^Tc‐sestamibi nuclear medicine scans for detecting parathyroid adenomas is unknown in horses but is reported to be ~90% in humans^17^ and 17% in dogs.^18^ Successful employment of ^99m^Tc‐sestamibi scintigraphic scanning to detect parathyroid adenomas in affected horses has been reported in 13 of 16 (81%) published cases.^8^, ^9^, ^10^, ^11^, ^16^ As reported in other equine cases, ^99m^Tc‐sestamibi nuclear medicine scanning failed to identify an abnormal parathyroid gland in this mule.^8^ Explanations for failure of ^99m^Tc‐sestamibi to identify a cause for high PTH concentration in human patients include patient motion, ectopy, small glands (<500 mg), radionuclide uptake interference by comorbid thyroid gland neoplasia, and parathyroid carcinoma.^19^, ^20^ Even on necropsy, an enlarged parathyroid gland could not be identified in this mule; it is likely that extensive lymphadenopathy in the area complicated the examination.

Chronic lymphocytic leukemia and small lymphocytic lymphoma (SLL) are grouped as a single entity in the WHO classification schemes of hematopoietic neoplasms for both humans and horses.^21^, ^22^ This is partly because, morphologically, the neoplastic cells are identical. However, oncologists in human medicine classify the disease as CLL when a clonal peripheral blood lymphocytosis >5 × 10^3^ cells/μL is present for at least 3 months.^23^ The same is true in veterinary oncology, and for these reasons, this mule was diagnosed with B‐cell CLL. As in our case, lymphadenomegaly is commonly observed in patients with CLL.

Chronic lymphocytic leukemia is rare in equids and has never been reported in a mule. Chronic lymphocytic leukemia represents 2% of equine hematopoietic malignancies, and there are at least 16 cases in the literature.^22^, ^24^, ^25^, ^26^, ^27^, ^28^, ^29^ In reported cases that had immunophenotyping performed, 9 were T cell and 2 were B cell. These two previously reported B cell CLL cases progressed rapidly, which contrasts with the disease course in humans and dogs.^29^, ^30^, ^31^ Documented lymphocytosis was evident in this mule for at least 1 year, but further diagnostic tests initially were not pursued. Despite severe hypercalcemia and possible lymphoid malignancy, the untreated mule continued with relatively normal quality of life. The presumed slow clinical progression of CLL in this case is similar to that expected for other species.^31^

Furthermore, histopathological findings in our case implied that slow disease progression would have been expected. A previous study described a grading scheme based on mitotic rate in lymphoma samples from horses.^22^ Mean mitoses per single high‐power field were reported by lymphoma subtype using the following criteria (indolent grade = 0‐1 mitoses, low grade = 2‐5 mitoses, mid‐grade = 6‐10 mitoses, and high grade = >10 mitoses). The mitotic index (13 mitoses in 10 high power [400×] fields) in this mule was consistent with indolent or low‐grade disease.

Immunophenotyping methods have evolved over the last 20 years, and diagnostic tests such as immunohistochemistry, immunocytochemistry, PARR, flow cytometry and sequencing have improved our ability to subcategorize lymphomas and leukemias.^32^, ^33^ Lymphocytes in this mule were characterized by flow cytometry and expressed CD44 (91%) and CD21 (66%), most consistent with B cell CLL. Polymerase chain reaction for antigen receptor rearrangement also was performed to confirm clonal expansion of B cells in lymph nodes.^32^

With concurrent hypercalcemia and CLL in this case, there was concern for paraneoplastic hypercalcemia, but PTHrP concentration was normal whereas PTH concentration was increased.^34^ Parathyroid hormone may be secreted by neoplastic tissue, and thus immunohistochemical staining for PTH was performed on neoplastic lymph nodes.^34^, ^35^, ^36^, ^37^ No stain uptake was noted. It therefore was concluded that ectopic parathyroid tissue was present, albeit not identified in this case.

A limitation of our case report is that the antibodies used for flow cytometry and immunohistochemistry have been validated in the horse, but their performance in a mule is unknown. Ideally, tissues from a control healthy mule would have been evaluated concurrently, but such samples were not available. However, the concordant data obtained from flow cytometry, immunohistochemistry (using different B cell markers and antibody clones) and PARR is compelling evidence that the assays were reliable for the diagnosis of a B cell neoplasm in this mule.

## 4 SUMMARY

Ours is the first reported case of CLL in a mule, and concurrent PHPT was particularly interesting. The B cell CLL in this case progressed slowly, which is contrary to previous reports of the same disease in horses but similar to the clinical course in dogs and humans. Hyperparathyroidism can present concurrently with neoplasia and should be ruled out as a differential diagnosis in equids with cancer.

## CONFLICT OF INTEREST DECLARATION

Authors declare no conflict of interest.

## OFF‐LABEL ANTIMICROBIAL DECLARATION

Authors declare no off‐label use of antimicrobials.

## INSTITUTIONAL ANIMAL CARE AND USE COMMITTEE (IACUC) OR OTHER APPROVAL DECLARATION

Authors declare no IACUC of other approval was needed. Owner permission was given for publication of this case report.

## HUMAN ETHICS APPROVAL DECLARATION

Authors decline human ethics approval was not needed for this study.
